# Polymorphisms in the TGFB1 and IL2RA genes are associated with clinical forms of leprosy in Brazilian population

**DOI:** 10.1590/0074-02760180274

**Published:** 2018-12-10

**Authors:** Rodrigo Mendes de Camargo, Weber Laurentino da Silva, Priscila Medeiros, Andrea de Faria Fernandes Belone, Ana Carla Pereira Latini

**Affiliations:** 1 Instituto Lauro de Souza Lima, Bauru, SP, Brasil; 2 Universidade Estadual Paulista Júlio de Mesquita Filho, Faculdade de Medicina de Botucatu, Departamento de Doenças Tropicais e Diagnóstico por Imagem, Botucatu, SP, Brasil

**Keywords:** leprosy, genetic epidemiology, TGFB1, IL2RA, Tregs

## Abstract

BACKGROUND Leprosy is a chronic infectious disease caused by *Mycobacterium leprae*, and compromises the skin and peripheral nerves. This disease has been classified as multibacillary (MB) or paucibacillary (PB) depending on the host immune response. Genetic epidemiology studies in leprosy have shown the influence of human genetic components on the disease outcomes. OBJECTIVES We conducted an association study for *IL2RA* and *TGFB1* genes with clinical forms of leprosy based on two case-control samples. These genes encode important molecules for the immunosuppressive activity of Treg cells and present differential expressions according to the clinical forms of leprosy. Furthermore, *IL2RA* is a positional candidate gene because it is located near the 10p13 chromosome region, presenting a linkage peak for PB leprosy. METHODS A total of 885 leprosy cases were included in the study; 406 cases from Rondonópolis County (start population), a hyperendemic region for leprosy in Brazil, and 479 cases from São Paulo state (replication population), which has lower epidemiological indexes for the disease. We tested 11 polymorphisms in the *IL2RA* gene and the missense variant rs1800470 in the *TGFB1* gene. FINDINGS The AA genotype of rs2386841 in *IL2RA* was associated with the PB form in the start population. The AA genotype of rs1800470 in *TGFB1* was associated with the MB form in the start population, and this association was confirmed for the replication population. MAIN CONCLUSIONS We demonstrated, for the first time, an association data with the PB form for a gene located on chromosome 10. In addition, we reported the association of *TGFB1* gene with the MB form. Our results place these genes as candidates for validation and replication studies in leprosy polarisation.

Leprosy is a chronic infectious disease caused by *Mycobacterium leprae*, an intracellular pathogen with a predilection for skin macrophages and Schwann cells, causing skin lesions and compromising peripheral nerves. It represents a significant public health problem, where approximately 210,000 new cases are diagnosed annually. Brazil ranks second in the incidence of leprosy globally.^1^

The disease presents as a broad clinical spectrum between two poles, tuberculoid (TT) and lepromatous (LL), based on a predominance of the Th1 or Th2 immune response, respectively. The TT pole represents the localised form of the disease with the highest bacillus containment, and the LL pole represents the disseminated form with the highest bacillary spread. In addition, there are three intermediate forms: borderline tuberculoid (BT), borderline borderline (BB), and borderline lepromatous (BL). This classification, proposed by Ridley and Jopling,^2^ is widely used in research centres and covers clinical, immunological, microbiological, and histopathological aspects. However, for treatment purposes, the World Health Organization (WHO) has classified leprosy into paucibacillary (PB) and multibacillary (MB) since 1982. The first WHO classification considered PB as TT and BT patients with a 1+ bacilloscopic index.^3^ Subsequently, WHO classified the PB cases only as those with a negative smear.^4^ Finally, in 1994, WHO classified PB and MB based on the number of lesions.^5^

Although several studies have demonstrated the participation of host genetic components in leprosy *per se* development, few studies have investigated the markers associated with the clinical forms of the disease. The number of genes associated with leprosy subtypes or its polarisation (39 associations in 28 genes) is lower than to leprosy *per se* (82 associations in 50 genes).^6^ In most cases, the association analyses of clinical forms are secondary to studies about leprosy per se and are often comparisons of clinical form groups to healthy controls. However, as discussed by Gaschignard and colleagues,^6^ these designs inappropriately consider the PB and MB forms of leprosy as “distinct diseases”. Further, the mechanisms involved in the disease per se are not necessarily involved in the determination of the clinical form.^6^ In addition, a two-stage model has been proposed to assess genetic susceptibility to leprosy, which includes a set of genes to determine the risk of leprosy per se and another set to determine the clinical form of leprosy.^7^

The following genes were determined to have replicated and/or validated associations with clinical forms of leprosy: 1) *TNF*, associated with the MB form in Indian^8^ and Thai populations,^9^ 2) *MRC1* and *MBL2*, associated with the MB form in Brazil^10^ ^,^ ^11^ and Nepal,^12^ and to the PB form in China,^13^ ^,^ ^14^ 3) *TLR2*, associated with the PB form in Ethiopia^15^ and Malawi and 4) *LRRK2* associated with the PB form in China^16^ and India.^17^ Besides, several studies have produced replicated association data for clinical forms of leprosy in the Chinese population.^13^ ^,^ ^14^ ^,^ ^18^ ^,^ ^19^ ^,^ ^20^ ^,^ ^21^ ^,^ ^22^ ^,^ ^23^ From linkage scans in two studies, one peak at chromosomal region 10p13 for PB leprosy was discovered.^24^ ^,^ ^25^

The *IL2RA* gene, which encodes the alpha subunit of the IL-2 receptor, is known as CD25. It is a functional and positional candidate gene for the clinical forms of leprosy as it is located at the chromosomal region 10p15, near the linkage peak for PB leprosy.^24^ ^,^ ^25^ CD25 is also an important marker of regulatory T cells (Tregs), which regulate the induction and maintenance of immunosuppression in lepromatous leprosy.^26^ High numbers of CD25+ cells are found in patients of the lepromatous pole and are associated with *M. leprae* proliferation.^27^ ^,^ ^28^ The nature of FOXP3 activity, whether inhibiting or activating, appears to differ when comparing leprosy poles.^29^ In addition, in lepromatous patients, miR155, which is involved in higher proliferation and longevity of Tregs,^29^ is seen to be overexpressed. Notably, Treg cells use this receptor to consume IL-2 at the site of the immune response, which hinders activation and proliferation of effector T lymphocytes and prevents activation of macrophages.^30^

The TGF-β is a pleiotropic cytokine, and it is an important effector molecule for the immunoregulatory activity of Tregs.^31^ It plays an important role in diseases caused by intracellular microorganisms, such as *Leishmania, Trypanosoma cruzi, Toxoplasma gondii, Lacazia loboi*, and mycobacteria, as it suppresses macrophage activation.^32^ In vitro and in situ studies confirm higher TGF-β production in lepromatous patients, which contributes to the anti-inflammatory milieu and bacillary persistence observed at this pole.^32^ ^,^ ^33^ ^,^ ^34^ ^,^ ^35^

Applying the strategy proposed by Gaschignard and researchers,^6^ we conducted an association study of *IL2RA* and *TGFB1* candidate genes in the “leprosy polarisation” phenotype, as this is a needy focus of inquiry in genetic epidemiology of leprosy.

## SUBJECTS AND METHODS

*Subjects and study design* - We applied a step-wise strategy based on two Brazilian cohorts. We investigated eleven markers at *IL2RA* gene and one marker at *TGFB1* gene in a start population. Then, the markers reaching Bonferroni-corrected p-value threshold significance were tested in the replication population.

Our start population was sampled from Rondonópolis County, located at Mato Grosso state, a hyperendemic region for leprosy in Brazil. The replication population is from the state of São Paulo, where the epidemiological indices of leprosy are more controlled.^36^

To classify patients as PB and MB, we adopted the WHO classification criterion of 1982.^3^ Taking into account the Ridley and Jopling^2^ spectrum, TT and BT patients with ≤ 1+ bacilloscopic indices were classified as PB, while those with bacilloscopic indices ≥ 2+ were classified as MB.

Patients from Rondonópolis included 406 individuals, of which 90 were PB and 310 MB. Leprosy diagnosis was confirmed by clinical laboratory tests in the outpatient service of the local family health clinics. São Paulo state enrolled 479 patients diagnosed at the Lauro de Souza Lima Institute (Bauru SP), of which 99 were PB and 380 were MB.

As a control for ethnicity in the association study, we defined the molecular ancestry for the start population by employing 46 ancestry informative indels, as previously described.^37^ The estimates of individual ancestry, European, African, and Native American were analysed using the ADMIXTURE software.^38^ The admixture fraction mean values were 0.58, 0.27, and 0.15 in cases and 0.58, 0.25, and 0.17 in controls for European, African, and Native American ancestries, respectively. The descriptions of the general characteristics of these groups are detailed in the Table I.

*DNA extraction and SNP genotyping* - Genomic DNA was extracted from peripheral blood leukocyte samples using the salting-out method. Genotyping was performed by the allelic discrimination method based on TaqMan® technology (Applied Biosystems, Foster City, CA, USA) and was carried out using the Step One Plus real-time PCR equipment (Applied Biosystems, Foster City, CA, USA).

*Markers selection* - The rs1800470 polymorphism of the *TGFB1* gene was selected based on the data from the literature, which reported the functional effects of this variant.^39^ ^,^ ^40^ ^,^ ^41^ ^,^ ^42^ ^,^ ^43^ ^,^ ^44^ ^,^ ^45^ ^,^ ^46^ ^,^ ^47^ This is a missense polymorphism, which promotes a proline to leucine substitution at the 10th residue.

For the *IL2RA* gene, 11 tag SNPs were selected from the International HapMap Project database, taking the minimum minor allele frequency of 0.1 and an r^2^ cut-off of 0.8 in the Yoruba population as parameters.^48^ The following SNPs were selected: rs7910961, rs11256497, rs12722561, rs2245675, rs2386841, rs3134883, rs4749926, rs6602392, rs706778, rs942201, and rs9663421.

*Statistical analyses* - The comparisons of allele, genotype, and carrier frequencies were performed using an univariate logistic regression model, with and without adjustment for the sex, as previously described.^49^ For the start population, we also used the molecular ancestry as a covariate in the regression model. From the indels data, we have employed ADMIXTURE software to estimate European, African, and Native American ancestries.^38^ So, we used these data for a continuous adjustment since there is not a consensus on the use of these continuous variables to classify ethnicity as a categorical variable. All analyses were performed using the statistical software R for Windows, version 2.5.1, and the package Genetics.

**TABLE I:** Characteristics of multibacillary leprosy (MB) and paucibacillary leprosy (PB) groups in the start and replication populations

Variable	Category	Start population (n = 406)	Replication population (n = 479)
Clinical form (WHO, 1982)	Paucibacillary Multibacillary	96 (24%) 310 (76%)	99 (21%) 380 (79%)
Clinical form (Ridley & Jopling, 1966)	LL BL BB BT TT IL	21 (5.1%) 63 (15.3%) 79 (19.2%) 156 (38.7%) 60 (14.6%) 27 (6.6%)	102 (21.2%) 131 (27.3%) 132 (27.5%) 49 (10.2%) 62 (12.9%) 3 (0.6%)
Age (mean ± SD) MB Age (mean ± SD) PB		42.9 ± 16.1 39.1 ± 16.1	37.9 ± 18.01 40.2 ± 18.01
Sex MB	Male Female	201 (65%) 109 (35%)	271 (71%) 109(29%)
Sex PB	Male Female	45 (47%) 51 (53%)	63 (64%) 36 (36%)

BB: bordeline bordeline leprosy; BL: borderline lepromatous leprosy; BT: borderline tuberculoid leprosy; IL: indeterminate leprosy; LL: polar lepromatous leprosy; SD: standard deviation; TT: polar tuberculoid leprosy.

In order to avoid the multiple comparisons effect, Bonferroni correction was adopted before testing the markers in the replication population.

To evaluate signal independence for markers at *IL2RA*, we measured linkage disequilibrium (LD) using the Haploview software, version 4.2.^50^ We used the Solid Spine of LD algorithm to define the blocks, using a D-value cut-off of 0.8.

The call rate > 0.95 was adopted as the quality control parameter, and all the polymorphisms met these criteria.

## RESULTS

*3.1. SNP rs2386841 of the IL2RA gene is associated with PB leprosy* - Eleven markers at the *IL2RA* gene were tested in the start population, and rs2386841 and rs6602392 markers presented positive signal (Table II, Supplementary data).

The LD plot for these eleven *IL2RA* markers revealed four blocks of LD and two singleton SNPs (Figure). To test SNPs in the replication population, we applied the Bonferroni correction as described by Duggal and colleagues,^51^ since SNPs at the same block are not considered independent. Thus, considering four models (two genotypic, one allelic and one carrier of minor allele) and six hypotheses (four haplotype blocks and two independent markers), the p-value threshold for *IL2RA* gene was found to be 0.002.

The adjusted data for the AA genotype of the rs2386841 [Odds ratio (OR): 5.45, Confidence interval (CI) 95%: 1.93-15.3, p-value: 0.0013] marker remained significantly associated with the PB leprosy after correction. Further, it was tested in the replication population, according to the step-wise strategy. However, this association was not replicated in the replication population, as described in Table II.

For the carriers of allele A at the rs6602392 marker, the p-value of 0.0271 did not reach significance after correction, and it was not tested in the replication population.

*SNP rs1800470 of the TGFB1 gene is associated with MB leprosy* - For the start population, the rs1800470 marker in the *TGFB1* gene was tested. The adjusted data demonstrated an association of the AA genotype (OR: 2.81, CI95%: 1.27-6.24, p-value: 0.0107) with the MB form of the disease. This association persisted even after applying Bonferroni correction, following the same strategy as for *IL2RA* gene (p-value < 0.0125), and this data was replicated in the replication population. In addition, the carriers of the A allele showed associations with the MB form in the replication population, as described in Table III.

**Figure:**
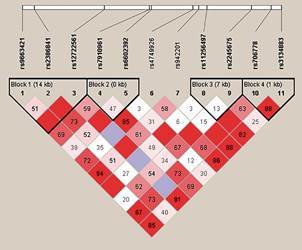
(Linkage disequilibrium LD) map for eleven SNPs at *IL2RA* gene genotyped for the start population. The number within boxes represents D-values calculated by Haploview software (4.2). The blocks were defined by Solid Spine of LD algorithm.

**TABLE II:** Frequency data in paucibacillary leprosy (PB) and multibacillary leprosy (MB) groups and association data for the markers rs2386841 and rs6602392 at the *IL2RA* gene

Population/marker	Alelles or genotypes	PB	MB	OR (95%CI) p-value	OR (95%CI) p-value^*a*^
Start population rs2386841	C A CC	0.76 0.24 59 (0.62)	0.83 0.17 214 (0.70)	* 1.57 (0.89-2.75) 0.1150 *	* 1.80 (0.98-3.27) 0.0546 *
	AC	27 (0.28)	83 (0.27)	1.17 (1.37-9.54) 0.5338	1.23 (0.70-2.17) 0.4613
	AA	9 (0.09)	9 (0.03)	3.62 (1.37-9.54) 0.0091	5.45 (1.93-15.3) 0.0013
	Carrier A			1.41 (0.87-2.29) 0.1538	1.57 (0.93-2.64) 0.0860
		n = 95	n = 306		
Start population rs6602392	C A CC AC AA **Carrier A**	0.89 0.11 74(0.78) 21(0.22) 0	0.82 0.18 209(0.68) 86(0.28) 11(0.04)	* 0.55 (0.26-1.16) 0.1189 * 0.66 (0.37-1.17)0.1626 - 0.58(0.33-1.03)0.0643	* 0.49 (0.23-1.05) 0.0669 * 0.60(0.33-1.07)0.0873 - **0.52(0.29-0.92)0.027**1
		n = 95	n = 306		
Replication population rs2386841	C A CC	0.76 0.24 44 (0.59)	0.74 0.26 175 (0.58)	* 0.91 (0.50-1.65) 0.7683 *	* 0.90 (0.49-1.62) 0.7322 *
	AC	24 (0.32)	97 (0.32)	0.98 (0.56-1.71) 0.9548	0.96 (0.54-1.67) 0.8889
	AA	6 (0.08)	30 (0.10)	0.79 (0.31-2.02) 0.6321	0.78 (0.30-2.00) 0.6133
	Carrier A			0.93 (0.56-1.57) 0.8131	0.91 (0.54-1.54) 0.7517
		n = 74	n = 302		

Bold values denote statistically significant results.*: indicates the baseline for comparison; *a*: Odds ratio (OR) e p-value adjusted for covariates sex and individual ancestry for start population and sex for replication population; CI: confidence interval. Global p-values (general test): rs2386841 - start population (p = 0.02), rs6602392 - start population (p = 0.02).

A combined analysis of both the populations, with adjustment for sex and origin covariates, was conducted. From this analysis, the association of the AA genotype with the MB form was confirmed (OR: 2.23, CI95%: 1.35-3.66, p-value: 0.0016). In addition, the A allele, and A carriers were associated with the MB form, as described in Table III.

## DISCUSSION

Following tests of 11 polymorphisms covering the *IL2RA* gene, we found that the AA genotype of rs2386841 was associated with susceptibility to the PB form in the start population. This is the second loci near the 10p13 region, associated with the PB form, which is supported by the findings from two linkage studies.^24^ ^,^ ^25^ Notably, the first associated gene, *MRC1*, had markers associated with PB leprosy in China, while it was associated with the MB form in Brazil and Vietnam.^10^ ^,^ ^13^ Moreover, the other associations described in this region, at *NEBL* and *CUBN* genes, were also related to the MB form.^52^ Thus, our data inserted a new candidate gene at this region, associated with the polarisation phenotype outcome.

Polymorphisms at the *IL2RA* gene are associated with lesion development in cutaneous leishmaniasis in Brazilian populations.^53^ In addition, the authors demonstrated a functional role for the associated alleles by decreasing the IFN-γ response and the Treg cell activities, which are closely related to the leprosy manifestations. Our results, coupled with Oliveira and colleagues, provide strong evidence of the importance of the *IL2RA* gene for the severity of infectious diseases caused by intracellular parasites.^53^ In order to reinforce this role, a relevant meta-analysis confirmed *IL2RA* as a susceptibility gene for Crohn’s Disease, which shares genetic risk factors with leprosy.^54^ ^,^ ^55^

When testing the *TGFB1* gene, we found an association between the AA genotype of the polymorphism rs1800470 to the risk of MB leprosy in both populations. The combined analysis with adjustments reinforced this association effect. This variant, also known as +29C> T, is located at the hydrophobic core of the signal peptide sequence; however, both alleles encode non-polar amino acids.^47^ Our findings are in conflict with the functional data, suggesting that the G allele is associated with higher production of TGF-β1^47^ since MB patients have higher levels of this cytokine.^34^ ^,^ ^35^ *In vitro* studies using different infection ratios as stimuli and considering the different genotypes for rs1800470 may be an interesting strategy to better explain this genetic data in leprosy polarisation.

**TABLE III:** Frequency data in paucibacillary leprosy (PB) and multibacillary leprosy (MB) groups and association data for the marker rs1800470 at the *TGFB1* gene

Population	Alelles or genotypes	PB	MB	OR (95%CI) p-value	OR (95%CI) p-value^*a*^
Start population	G	0.55	0.45	*	*
	A	0.45	0.55	1.49 (0.92-2.41) 0.0999	1.57 (0.94-2.63) 0.0796
	GG	24 (0.27)	54 (0.19)	*	*
	AG	50 (0.56)	146 (0.52)	1.29 (0.72-2.31) 0.3770	1.32 (0.72-2.43) 0.3545
	AA	15 (0.17)	83 (0.29)	2.45 (1.18-5.10) 0.0158	2.81 (1.27-6.24) 0.0107
	Carrier A			1.56 (0.89-2.72) 0.1127	1.63 (0.91-2.93) 0.0963
		n = 89	n = 283		
Replication population	G	0.53	0.45	*	*
	A	0.47	0.55	1.40 (0.87-2.26) 0.1650	1.40 (0.87-2.27) 0.1637
	GG	25 (0.30)	68 (0.20)	*	*
	AG	39 (0.46)	174 (0.50)	1.64 (0.92-2.91) 0.0917	1.66 (0.93-2.96) 0.0845
	AA	20 (0.24)	106 (0.30)	1.94 (1.00-3.77) 0.0483	1.96 (1.00-3.81) 0.0472
	Carrier A			1.74 (1.01-2.98) 0.0424	1.76 (1.02-3.03) 0.0394
		n = 84	n = 348		
Combined populations	G	0.54	0.45	*	*
	A	0.46	0.55	1.45 (1.03-2.04) 0.0294	1.45 (1.03-2.04) 0.0306
	GG	49 (0.28)	122 (0.19)	*	*
	AG	89 (0.51)	320 (0.51)	1.44 (0.96-2.16) 0.0763	1.47 (0.97-2.22) 0.0636
	AA	35 (0.20)	189 (0.30)	2.16 (1.32-3.53) 0.0020	2.17 (1.32-3.57) 0.0020
	Carrier A			1.64 (1.12-2.42) 0.0110	1.67 (1.13-2.47) 0.0095
		n = 173	n = 631		

Bold values denote statistically significant results.*: indicates the baseline for comparison; *a*: Odds ratio (OR) e p-value adjusted for covariates sex and individual ancestry for start population; sex for replication population; sex and origin for combined populations; CI: confidence interval. Global p-values (general test): rs1800470 - start population (p = 0.02), rs1800470 - replication population (0.12), combined populations (p = 0.01).

According to the 2016 WHO data, 72% of new Brazilian leprosy cases were MB.^56^ Therefore, 76% of MB cases in our both study populations reflect the leprosy epidemiology in Brazil. Despite similar distributions, we observed major representation of the BL and LL categories in the replication population and overrepresentation by the BT form in the start population. This is a limitation of our study, but it can help explain the replication of the *TGFB1* association with the MB form and the non-replication of the *IL2RA* association with the PB form. This also reinforces the necessity for more investigations focusing on the *IL2RA* gene.

Classical variables influencing the outcome of clinical forms of leprosy are sex, endemicity, geography, age, and BCG vaccination.^6^ To control for biases, we adopted selection criteria and analysis parameters to account for the effects of these covariates. Although ethnicity is not a classical risk factor influencing clinical forms of leprosy, human genetic factors influence the effect associated to the geography and endemicity. Thus, we considered ethnicity in our analysis due to the diverse admixture of races in Brazil, relative to the region of the country. Although the ancestry data revealed no substantial distortion between cases and controls, they were used to adjust the analysis in order to control some bias due to this variable in our study. Data from the literature indicated a higher incidence of the MB form in men than in women, with a ratio of 1.5-2.^6^ The number of MB individuals is also higher in Brazilian men than women.^56^ Our population samples followed this observation since the male: female ratios in the MB groups were 1.84 for the start population and 2.48 for the replication population. When adjusting our analysis using sex and ancestry data as covariates, we observed a stronger association effect for rs2386841 at *IL2RA*. This confirms the importance of considering these covariates when studying leprosy polarisation outcome.

A higher mean age of MB, relative to PB patients has also been reported, which is likely due to the long incubation time of the bacillus.^56^ ^,^ ^57^ ^,^ ^58^ Although the influence of age is higher in men than in women, the effect of these covariates (age and sex) are independent in leprosy polarisation.^58^ In our study, the mean age of illness for both populations is consistent with observations from the Brazilian population, which has a predominance of adult cases.^56^ Moreover, we did not observe relevant differences in age between PB and MB cases.

Leprosy is a complex trait and factors related to the host, environment, and pathogen act in the development of the disease per se and its clinical forms.^7^ However, there is no evidence that different strains of *M. leprae* interfere in the leprosy outcome; also, studies suggest low genetic variability of the bacillus.^59^ Therefore, considering the broad clinical spectrum of leprosy, the human genetic component seems to play a more relevant role in the outcome of the disease.^7^ From the epidemiological perspective, clarifying which host genetic factors are involved in leprosy polarisation may help predict clinical forms with higher potential to transmit disease, thus helping interrupt the transmission chain. As a result, our data points to *IL2RA* and *TGFB1* associations with leprosy polarisation and help to construct the genetic architecture of this neglected phenotype.
