# Supplementary material for: Polymorphisms in the TGFB1 and IL2RA genes are associated with clinical forms of leprosy in Brazilian population
Source: Mem Inst Oswaldo Cruz. 2018 Dec 10;113(12):e180274. doi: 10.1590/0074-02760180274 (PMC6287188; doi:10.1590/0074-02760180274)
Supplement: Supplementary file 1 [file 1678-8060-mioc-113-12-e180274-s.pdf]

TABLE

Frequency data in paucibacillary leprosy (PB) and multibacillary leprosy (MB) groups and association data for markers rs11256497, rs12722561, rs2245675, rs3134883, rs4749926, rs706778, rs942201, rs9663421 and rs7910961 at the *IL2RA* gene

Population/ marker	Alelles or genotypes	PB	MB	OR (95%CI) p-value	OR (95% CI) p-value[a]
Start population	G	0.72	0.65	*	*
rs11256497	A	0.28	0.35	0.71 (0.42-1.21) 0.2158	0.72 (0.42-1.23) 0.2325
	GG	51 (0.54)	133 (0.43)	*	*
	GA	36 (0.38)	135 (0.44)	0.66 (0.39 - 1.11) 0.1188	0.61 (0.26-1.42)0.1001
	AA	8 (0.08)	38 (0.12)	0.58 (0.25 - 1.34) 0.2041	0.61 (0.26-1.42)0.2536
	Carrier A			0.64(0.39-1.04)0.0760	0.63(0.39-1.04)0.0722
Start population	G	0.87	0.90	*	*
rs12722561	A	0.13	0.10	1.31 (0.63-2.73) 0.4558	1.28 (0.61-2.69) 0.5067
	GG	71(0.75)	242(0.79)	*	*
	GA	22(0.23)	62(0.20)	1.19 (0.66-2.15) 0.5564	1.16(0.63-2.11)0.6223
	AA	2(0.02)	2(0.01)	3.45 (0.47- 24.9) 0.2195	3.26(0.43-24.5)0.2507
	CarrierA			1.27(0.71-2.25)0.4073	1.23(0.69-2.21)0.4704
Start population	G	0.57	0.60	*	*
rs2245675	A	0.43	0.40	1.11 (0.68-1.80) 0.6743	1.17 (0.71-1.93) 0.5167
	GG	33(0.35)	114(0.37)	*	*
	GA	44(0.46)	138(0.45)	1.17 (0.68-2.00)0.5636	1.20 (0.69-2.09)0.4963
	AA	18(0.19)	54(0.18)	1.18(0.60-2.34)0.6201	1.34(0.66-2.69)0.4076
	Carrier A			1.17(0.71-1.94)0.5256	1.24(0.74-2.07)0.3999
Start population	G	0.82	0.82	*	*
rs3134883	A	0.18	0.18	1.01 (0.54-1.90) 0.9535	0.96 (0.50-1.8) 0.9104
	GG	67 (0.71)	208 (0.68)	*	*
	GA	24 (0.25)	88 (0.29)	0.90(0.52-1.56)0.7256	0.81(0.46-1.43)0.4755
	AA	4 (0.04)	10 (0.03)	1.43(0.42-4.83)0.5579	1.50(0.43-5.18)0.5183
	Carrier A			0.95(0.57-1.61)0.8752	0.87(0.51-1.49)0.6301
Start population	G	0.53	0.57	*	*
rs4749926	A	0.47	0.43	1.14 (0.71-1.87)0.5572	1.17 (0.72-1.92) 0.5100
	GG	28 (0.29)	103 (0.34)	*	*
	GA	44 (0.46)	141 (0.46)	1.22(0.70-2.14)0.4678	1.28(0.72-2.26)0.3916
	AA	23(0.24)	62 (0.20)	1.29(0.66-2.51)0.4527	1.33(0.67-2.63)0.4037
	Carrier A			1.24(0.74-2.10)0.4022	1.29(0.76-2.20)0.3335
Start population	G	0.59	0.57	*	*
rs706778	A	0.41	0.43	0.93 (0.57-1.52) 0.7913	0.91 (0.55-1.50) 0.7358
	GG	33 (0.35)	105 (0.34)	*	*
	GA	45 (0.47)	140 (0.46)	0.98(0.57-1.67)0.9420	0.96(0.55-1.66)0.8912
	AA	6 (0.18)	61 (0.20)	0.87(0.43-1.73)0.6954	0.84(0.41-1.68)0.6246
	Carrier A			0.94(0.57-1.56)0.8307	0.92(0.55-1.54)0.7639
Start population	C	0.86	0.86	*	*
rs942201	A	0.14	0.14	1.07 (0.53-2.13) 0.8385	1.08 (0.53-2.18) 0.8212
	CC	72 (0.76)	230 (0.75)	*	*
	AC	20 (0.21)	67 (0.22)	1.03(0.57-1.85)0.9081	1.02(0.56-1.85)0.9384
	AA	3 (0.03)	8 (0.03)	1.24(0.32-4.83)0.7503	1.35(0.33-5.38)0.6705
	Carrier A			1.05(0.60-1.84)0.8380	1.05 (0.60-1.86)0.8427
Start population	G	0.71	0.70	*	*
rs9663421	A	0.29	0.30	0.97 (0.57-1.65)0.9268	0.97 (0.57-1.66) 0.9315
	GG	46 (0.48)	153(0.50)	*	*
	GA	40 (0.42)	121 (0.40)	1.13(0.68-1.87)0.6246	1.16(0.69-1.95)0.5524
	AA	9(0.09)	32 (0.10)	0.78(0.32-1.91)0.5976	0.76(0.30-1.88)0.5593
	Carrier A			1.05(0.65-1.71)0.8126	1.07(0.66-1.76)0.7621
Start population	G	0.57	0.65	*	*
rs7910961	A	0.43	0.35	1.39 (0.85-2.26) 0.1861	1.45 (0.88-2.40) 0.0972
	GG	32(0.34)	134(0.44)	*	*
	AG	43(0.45)	127(0.42)	1.43(0.83-2.46)0.1883	1.54(0.88-2.67)0.1236
	AA	20(0.21)	45(0.15)	1.77(0.89-3.49)0.0986	1.91(0.95-3.83)0.0670
	CarrierA			1.52(0.92-2.51)0.0969	1.64(0.98-2.74)0.0577
		n = 95	n = 306		

*: indicates the baseline for comparison; *a*: OR (Odds ratio) e p-value adjusted for covariates sex and individual ancestry; CI: confidence interval.

CC BY
